# The Health Policy Attitudes of American Medical Students: A Pilot Survey

**DOI:** 10.1371/journal.pone.0140656

**Published:** 2015-10-16

**Authors:** Robert A. Dugger, Abdulrahman M. El-Sayed, Catherine Messina, Richard Bronson, Sandro Galea

**Affiliations:** 1 Columbia University Mailman School of Public Health, New York, New York, United States of America; 2 Stony Brook University School of Medicine, Stony Brook, New York, United States of America; 3 Columbia University College of Physicians and Surgeons, New York, New York, United States of America; 4 Hofstra North Shore Long Island Jewish School of Medicine, Queens, New York, United States of America; Duke University Medical Center, UNITED STATES

## Abstract

**Background:**

Relatively little is known about American medical student’s attitudes toward caring for the uninsured, limiting physician reimbursement and the role of cost-effectiveness data in medical decision-making. We assessed American medical student’s attitudes regarding these topics as well as demographic predictors of those attitudes, and compared them to practicing physicians.

**Methods and Findings:**

A survey instrument was explicitly designed to compare medical student attitudes with those previously reported by physicians. Between December 1st 2010 and March 27th 2011 survey responses were collected from more than 2% of the total estimated 2010–2011 US medical student population enrolled at 111 of 159 accredited US medical schools within the 50 United States (n = 2414 of possible 98197). Medical students were more likely to object to reimbursement cuts, and more likely to object to the use of cost effectiveness data in medical decision making than current physicians according to the literature. Specialty preference, political persuasion, and medical student debt were significant predictors of health policy attitudes. Medical students with anticipated debt in excess of $200,000 were significantly less willing to favor limiting reimbursement to improve patient access (OR: 0.73 [95% confidence interval (CI): 0.59–0.89]), and significantly more likely to object to using cost effectiveness data to limit treatments (OR 1.30, 95% CI 1.05–1.60) when compared to respondents with anticipated debt less than $200,000.

**Conclusions:**

When compared to physicians in the literature, future physicians may be less willing to favor cuts to physician reimbursements and may be more likely to object to the use of cost effectiveness data. Political orientation, specialty preference and anticipated debt may be important predictors of health policy attitudes among medical students. Early career medical providers with primary care ambitions and those who anticipate less debt may be more likely to support healthcare cost containment.

## Introduction

Physicians are important stakeholders in American healthcare [1]. In that respect, their attitudes toward important healthcare policy questions, such as the obligation to care for the uninsured, support for expanding patient access via limiting physician reimbursement, and the role of cost-effectiveness data in medical decision-making, may have important implications for national health policy decisions. For example, prior to the enactment of the Patient Protection and Affordable Care Act, which was forecasted to decrease substantially the proportion of uninsured Americans, a majority of surveyed American physicians supported the expansion of healthcare coverage to the under- and uninsured at the expense of physician reimbursements. This suggests that a majority of physicians were willing to receive less compensation for their services if that translated into greater patient access to health care [2]. A study by Antiel et al. also assessed physician perspectives on the use of cost effectiveness data in determining treatments for patients. As the term implies, cost-effectiveness approaches take into account the relative cost of treatment in addition to comparative clinical-effectiveness when allocating treatments [3]. Utilizing cost-effectiveness in treatment may be controversial, as 54% of physicians in the study by Antiel et al. reported moral objections to its use [2].

Investigation into the health policy attitudes of American physicians is not limited to doctors currently in practice [4–8]. In 2011 Huntoon et al. suggested that, at 10 medical schools, a majority of medical students supported the Patient Protection and Affordable Care Act (80%). However, these students echoed concerns about whether the Patient Protection and Affordable Care Act would adequately address issues of quality or cost containment [7]. Subsequent study of medical student policy beliefs further highlights support for the affordable care act, however, the assessment and analysis of critical cost-containment approaches therein is limited [9–15]. This is a particular limitation with respect to understanding potential facilitators, or barriers, for future cost conscious health policy as medical students may have interests that differ from those of current physicians.

Medical students may have substantially different policy preferences than do practicing physicians for several reasons. First, medical students are one or two generations younger than practicing physicians, with opinions informed by changing temporal trends in cultural norms and attitudes. Second, current students’ formative training has occurred during notable polarization around the future of healthcare policy. Third, medical students may be less willing to take cuts in reimbursement given the rising student debt burden, which has quadrupled over the past few decades [16]. For example, the Association of American Medical Colleges reports the median medical student educational debt as $200,000 in 2014 [17]. Fourth, medical students are not yet specialized, and therefore, they may be less aligned to the particular economic interests of medical specialties.

Within a sample comprising over two percent of American medical students from among 111 of 159 American medical schools, we explored the health policy attitudes of American medical students with respect to the obligation to care for the under- and uninsured, limits on reimbursements for medications and procedures, and the use of cost-effectiveness analysis in medical decision-making using a survey designed to mirror a similar survey representative of practicing physicians in the United States [2]. We also considered the roles of anticipated educational debt, political orientation, and specialty preference as predictors of these attitudes.

## Methods

### Data

To obtain our sample, we did the following: First, representatives to the Organization of Student Representatives, American Medical Association, Council of Osteopathic Student Government Presidents and faculty deans at all 159 of the 2010–2011 accredited US medical schools (allopathic and osteopathic) within the 50 United States were mailed participation information (a total of 1183 contacts). Second, institutional contacts were asked to forward the secured consent forms and the confidential electronic surveys to their respective medical student bodies. Third, participants self-administered the surveys between December 1^st^ 2010 and March 27^th^ 2011. Respondents were entered into a drawing for a $100 gift card as an incentive.

Students from 111 institutions participated, providing a total of 2414 responses. Participants were excluded from the study if their responses were incomplete, they provided duplicate contact information for incentive distribution, they did not identify as a 1st, 2nd, 3rd or 4th year medical student (i.e., they were taking time off or pursuing a second degree), or did not attend a medical school in the US (S1 File, **Excluded Respondents**). Ninety-eight percent of the 2414 responses met study inclusion criteria (N = 2355).

Our survey instrument, described in detail elsewhere [18], was explicitly designed to compare medical student attitudes with those previously reported by physicians [2]. We conducted two cycles of cognitive interview pretesting with 9 and 7 medical students, respectively. We used the feedback from these sessions to revise the items for consistency and clarity (S2 File, **Survey Instrument**).

### Outcomes of Interest

Our outcomes of interest included three important policy attitudes considered in a previous study of US physicians [2]. Participants were asked to specify their agreement or disagreement with the following statements:

- “Every physician is professionally obligated to care for the uninsured and underinsured;”
- “I would favor limiting reimbursement for expensive drugs and procedures if that would help expand access to basic health care for those currently lacking such care.”

Responses were measured on a four-point Likert-type scale (Strongly Disagree, Moderately Disagree, Moderately Agree, Strongly Agree). We then asked medical students to indicate whether they had no moral objection, a moderate moral objection, or a strong moral objection to “using cost-effectiveness data to determine which treatments will be offered to patients.” This question, like the two prior attitudes of interest, is modeled from the question posed to physicians in the literature [2], and was designed by those authors to highlight points of genuine ambivalence or disagreement among physicians. Notably, the survey instruments intentionally refrained from educating respondents with term definitions or referral to other literature. Outcomes were analyzed in the form of binary agree/disagree (questions 1 and 2) and objection/no objection (question 3) responses for regression analysis to maximize statistical power.

### Predictors of interest

We considered a number of potential predictors of key attitude outcomes among medical students. First, respondents were asked their political self-characterization as “Conservative,” “Moderate,” “Liberal” or “Other.” Only five percent of the sample included those who self-reported their political orientation as “Other.” We considered systematic differences in 21 other covariates considered in our analysis across political self-characterization using chi-square analyses, and found substantial similarity between ‘conservative’ and ‘other’ respondents. Therefore, they were grouped accordingly (S3 File, **Analysis of Political Orientation**). Second, we considered future career aspirations within medicine which mirrored response categories from the comparator physician study (“Primary care,” “Surgery,” “Procedural Specialty,” “Nonprocedural specialty,” “Nonclinical specialty” and “Other”) with the addition of “Undecided” to accommodate students who had not yet decided upon a specialty. Third, anticipated educational debt upon graduation (No educational debt, Less than $100,000, $100,000–$150,000, $150,000–$200,000, $200,000–$250,000, $250,000–$300,000, Greater than $300,000) was assessed and analyzed as a binary variable denoting anticipated educational debt greater than or less than $200,000, which approximated the median anticipated educational debt in our sample and corresponded with average debt reported in the literature. Information was also collected about race/ethnicity (White, Black, Hispanic/Latino, Asian, Multiple/Other), gender (male, female), age (analyzed as a binary variable; <25 years of age, ≥ 25 years of age), clinical level in medical school (1^st^ year, 2^nd^ year, 3^rd^ year, 4^th^ year), category of medical school (e.g., public, private) and medical school region (South, Midwest, Northeast, West).

We weighted our sample to improve representativeness across the US medical student population. We collected data from the Association of American Medical Colleges and American Association of Colleges of Osteopathic Medicine about the population of medical students enrolled in Doctor of Medicine (MD) and Doctor of Osteopathic Medicine (DO) schools in the 2010–2011 academic year to weight our sample for representativeness by race and class year in medical school. Differences between these two data sets required relevant category responses to be collapsed as appropriate to facilitate compatibility across datasets [19–24].

### Analysis

First, we calculated univariate statistics to describe our sample as well as the responses on our outcomes of interest. Second, we performed chi-square analysis between exposures and outcomes to evaluate bivariate relationships. Third, we used multivariable logistic regression to determine whether student clinical specialty preference, political affiliation, or anticipated debt upon graduation after adjusting for demographic characteristics, were associated with responses to our three key outcome measures after adjustment for age, sex, race and region. All analyses accounted for weighting using SAS 9.3.

### Ethics statement

This study was approved by the Stony Brook Committees on Research Involving Human Subjects. All data underlying the study findings are freely available (S4 File, **Dataset**).

## Results

Table 1 shows respondent characteristics and responses. Seventy-three percent of medical students agreed that every physician is professionally obligated to care for the under- and uninsured. Sixty-three percent of medical students were willing to accept limits on reimbursement for expensive drugs and procedures in order to improve access to basic health care. Lastly, sixty-two percent of medical students objected to using cost effectiveness data to determine which treatments will be offered to patients. Nearly forty-three percent of respondents reported anticipated debt upon graduation in excess of $200,000 (not shown).

**Table 1:** Combined allopathic and osteopathic medical student demographics and outcome responses, 2010–2011.

Characteristic	Sample frequency	Weighted percent	Estimated percent in the medical student population*
Female Sex	1177	51.2	47.3
Self Described Race and or Ethnicity			
White	1752	57.9	59.9
Asian	322	21.3	21.2
Black	51	7.3	6.2
Hispanic/Latino	62	8.0	7.0
Other	168	5.5	5.6
Level of Medical School			
1st year	759	25.1	26.8
2nd year	737	24.7	25.5
3rd year	428	25.0	24.4
4th year	431	25.2	23.2
Category of Medical School			
Public	1052	45.7	50.9
Private	1303	54.3	49.1
Medical School Region			
South	335	15.1	32.4
Midwest	748	29.1	24.8
Northeast	668	29.5	28.6
West	604	26.4	14.2
Every physician is professionally obligated to care for the uninsured and the underinsured. P<0.001			
Strongly Agree	769	33.1	
Moderately Agree	944	40.0	
Moderately Disagree	413	17.3	
Strongly Disagree	229	9.5	
I would favor limiting reimbursement for expensive drugs and procedures if that would help expand access to basic health care for those currently lacking such care. P<0.001			
Strongly Agree	385	17.8	
Moderately Agree	1058	45.2	
Moderately Disagree	567	23.6	
Strongly Disagree	345	13.5	
Indicate the degree to which you object (if at all), for moral reasons to the following practice; Using cost-effectiveness data to determine which treatments will be offered to patients. P<0.001			
No Moral Objection	847	38.0	
Moderate Moral Objection	1141	47.6	
Strong Moral Objection	367	14.4	

*Data compiled from American Association of Medical Colleges and the American Association of Colleges of Osteopathic Medicine records of 2010–2011 total student enrollment. Percentages may not total 100 due to rounding. Self-Described Race and or Ethnicity accepted more than one answer for respondents and population MD students whereas data for DO students collapsed multiple races/ethnicities into the other category. Student population data includes levels other than 1st-4th year. Population data includes schools in Puerto Rico whereas the sample does not. MD student level population derived from the matriculating student questionnaire which also includes non-US accredited matriculation and also has augmented enrollment totals. Values rounded to one decimal place and therefore may not add up to 100.

Table 2 shows weighted chi-square analysis of relations between predictors of interest and health policy responses. With respect to obligation to care for the uninsured, we found that primary care career preference, age less than 25, female gender, pre-clinical medical school level, and public medical school category predicted higher likelihood of agreement. On the other hand, Caucasian race, southern medical school region and conservative political orientation predicted lower likelihood of agreement. There was no association between anticipated educational debt and agreement with obligation to care for the uninsured. With respect to agreement with limits to reimbursement, we found that primary care career preference, female gender, and western medical school region predicted higher likelihood of agreement. Conversely, anticipated debt greater than $200,000, Caucasian race, conservative political orientation and predicted lower likelihood of agreement. There was no association between agreement with limits to reimbursement and age, medical school level or medical school category. With respect to using cost-effectiveness data to limit treatments, we found that age greater than 25, male gender and public medical school category predicted less objection. Anticipated educational debt greater than $200,000 and Conservative political orientation and predicted greater objection. There was no association between race, medical school level, medical school region or specialty preference and objection to the use of cost-effectiveness data to help determine treatments.

**Table 2:** Weighted chi-square analysis of health policy principles, among 2355 U.S. medical students.*

Variable	Physicians Are Obligated to Care for the Underinsured			Limiting Reimbursement for Expensive Treatments to Expand Access to Basic Health Care			Using Cost-Effectiveness Data to Limit Treatments		
	Disagree	Agree	χ^2^ p	Disagree	Agree	χ^2^ p	No Moral Objection	Moral Objection	χ^2^ p
Age			<0.01			0.42			0.04
<25	21.1	78.9		38.3	61.7		41.3	58.7	
>24	29.6	70.4		36.4	63.6		36.3	63.7	
*Gender*			<0.01			<0.01			<0.01
Male	33.4	66.6		42.6	57.4		43.5	56.5	
Female	20.5	79.5		31.7	68.3		32.7	67.3	
*Race*			<0.02			<0.01			0.09
White	30.9	69.1		42.3	57.7		34.6	65.4	
Asian	21.2	78.8		30.0	70.0		43.6	56.4	
Black	20.9	79.1		22.0	78.0		45.4	54.6	
Hispanic/Latino	19.7	80.3		32.8	67.2		39.7	60.3	
Multiple/Other	24.6	75.4		34.8	65.2		38.9	61.1	
*Medical School Level*			<0.01			0.39			0.09
1st year	21.3	78.7		36.4	63.6		38.0	62.0	
2nd year	22.8	77.2		34.1	65.9		34.1	66.0	
3rd year	31.2	68.8		39.5	60.5		42.6	57.4	
4th year	32.0	68.0		38.1	61.9		37.1	62.9	
*Medical School Category*			<0.02			0.18			<0.01
Public	24.1	75.9		35.3	64.7		42.7	57.3	
Private	29.1	70.9		38.5	61.5		33.9	66.1	
*Medical School Region*			<0.02			<0.01			0.39
South	33.8	66.2		46.1	53.9		35.7	64.3	
Midwest	27.1	72.9		39.5	60.5		35.8	64.2	
Northeast	22.5	77.5		36.3	63.7		40.8	59.2	
West	27.4	72.6		29.9	70.1		38.5	61.5	
*Political Orientation*			<0.01			<0.01			<0.01
Moderate	25.6	74.4		37.4	62.6		39.7	60.3	
Liberal	16.8	83.2		21.8	78.2		40.4	59.6	
Conservative	49.4	50.6		67.2	32.8		29.8	70.2	
*Specialty Preference*			<0.01			<0.01			0.28
Primary Care	19.4	80.6		27.2	72.8		38.1	61.9	
Surgery	33.4	66.6		52.1	47.9		33.8	66.2	
Other Specialty/Discipline	33.5	66.5		42.1	57.9		37.8	62.2	
Undecided	19.8	80.2		32.3	67.7		42.9	57.1	
*Anticipated Educational Debt*			0.06			<0.01			<0.01
Less than $200,000	25.1	74.9		34.2	65.8		40.7	59.3	
Greater than $ $200,000	29.1	70.9		40.9	59.1		34.3	65.7	

*Values rounded and therefore may not add up to 100.

Table 3 shows the odds of each of our three outcomes by political orientation, specialty, and educational debt, adjusting for age, sex, race, and region. Both specialty preference and political orientation were associated with medical student outcome responses. Medical students preferring a career in surgery and those preferring careers in other specialties were significantly less likely to agree that physicians are obligated to care for the under- and uninsured when compared to medical students preferring a career in primary care (OR = 0.51, 95 CI 0.36–0.73 and OR = 0.47, 95 CI 0.36–0.61 respectively). Medical students preferring a career in surgery and those preferring a career in other specialties were also significantly less favorable to limiting reimbursement for expensive treatments to expand access to basic health care when compared to medical students preferring a career in primary care (OR = 0.37, 95 CI 0.26–0.52 and OR = 0.53, 95 CI 0.41–0.67 respectively).

**Table 3:** Weighted odds of endorsing health policy principles and of objecting to the use of cost-effectiveness data to limit treatments, according to clinical specialty preference, political self-characterization and level of anticipated debt upon graduation, among 2355 U.S. medical students.*

Variable	Agree That Physicians Are Obligated to Care for the Underinsured		Favor Limiting Reimbursement for Expensive Treatments to Expand Access to Basic Health Care		Object to Using Cost-Effectiveness Data to Limit Treatments	
	OR	95% CI	OR	95% CI	OR	95% CI
*Specialty Preference*						
Primary Care (Reference)						
Surgery	0.51	0.36–0.73	0.37	0.26–0.52	1.46	1.04–2.05
Other Specialty/Discipline	0.47	0.36–0.61	0.53	0.41–0.67	1.11	0.87–1.41
Undecided	0.91	0.65–1.27	0.82	0.59–1.13	0.95	0.69–1.31
*Political Orientation*						
Conservative (Reference)						
Moderate	2.57	1.97–3.35	3.12	2.40–4.06	0.66	0.51–0.87
Liberal	4.21	3.11–5.68	6.32	4.75–8.41	0.59	0.45–0.78
*Debt*						
Less Than $200,000 (Reference)						
Greater Than $200,000	0.87	0.70–1.10	0.73	0.59–0.89	1.3	1.05–1.60

* Odds ratios are from weighted multivariate logistic regression, with adjustment for age, sex, race, and region.

Respondents favoring a career in surgery were significantly more likely to object to using cost effectiveness data to help determine treatments (OR = 1.46, 95 CI 1.04–2.05). When compared to medical students indentifying as conservative, both self-described moderates and liberals were significantly more likely to agree that physicians are obligated to care for the under- and uninsured (OR = 2.57, 95 CI 1.97–3.35 and OR = 4.21, 95 CI 3.11–5.68 respectively); to favor limiting reimbursement to improve patient access (OR = 3.12, 95 CI 2.40–4.06 and OR = 6.32, 95 CI 4.75–8.41 respectively); and they were also significantly less likely to object to using cost effectiveness data to limit treatments (OR = 0.66, 95 CI 0.51–0.87 and OR = 0.59, 95 CI 0.45–0.78 respectively). Anticipated debt upon graduation was associated with health policy attitudes relating to cost and reimbursement. Medical students with anticipated debt in excess of $200,000 were significantly less willing to favor limiting reimbursement to improve patient access when compared to respondents with anticipated debt less than $200,000 (OR = 0.73, 95 CI 0.59–0.89). Medical students with anticipated debt in excess of $200,000 were also significantly more likely to object to using cost effectiveness data in medical decision-making when compared to respondents with anticipated debt less than $200,000 (OR = 1.3, 95 CI 1.05–1.60).

## Discussion

In a study of over two percent of the US medical student population, we explored the health policy attitudes of American medical students. We found that a large majority of medical students endorsed an obligation to care for the under- and uninsured. A sizable majority of medical students were also willing to take cuts in reimbursement to improve patient access. By contrast, nearly sixty-three percent of medical students were opposed to the use of cost-effectiveness data in determining patient treatment.

In comparing the healthcare attitudes of American medical students to their practicing counterparts, there are some similarities and differences. Medical students and physicians were equally likely to endorse a professional obligation to care for the under- and uninsured (73% for both students and physicians) [2]. This suggests that, despite increasing financial constraints in an uncertain healthcare climate, medical students have largely adhered to a professional ethos of service. By contrast, however, medical students were less favorable toward reimbursement cuts to support improvements in healthcare access compared to practicing physicians (62% of students favored limiting reimbursement vs 67% of physicians) [2]. This may reflect financial constraints resulting from the increase in debt burden associated with medical education [25]. This explanation is supported by our findings that high anticipated debt predicted lower likelihood of support for cuts in reimbursement to expand access to basic healthcare services for the under and uninsured.

Medical students were also more likely than physicians to object to the use of cost-effectiveness analyses in care allocation than their physician counterparts (62% of students had moral objections versus 54% of physicians) [2]. Objection was particularly high among medical students with anticipated debt upon graduation greater than $200,000. One potential explanation for this finding is that medical students may perceive cost-effectiveness approaches to healthcare as limiting of their potential future earnings, particularly considering that cost-effectiveness analyses are often less likely to support treatment modalities that are more common among lucrative specialties [3]. Our finding that medical students intending upon careers in surgery were nearly fifty percent more likely to object to the use of cost-effectiveness approaches than those intending upon careers in primary care is supportive in this regard. However, it is also plausible that medical students may have limited familiarity with cost-effectiveness approaches relative to physicians, which may contribute to their discomfort with such approaches to clinical decision-making [26].

Specialty preference, political orientation, and anticipated debt upon graduation were important predictors of medical student health policy attitudes. Medical students intent upon careers in primary care were more likely to endorse a professional obligation to care for the under- and uninsured, to favor limiting reimbursements to expand care to the underserved, and to favor cost-effectiveness approaches in care allocation. This suggests that important differences in student attitudes may influence career choices. Moreover, it challenges a commonly-held perception that medical students choosing careers in primary care or related specialties do so simply because they lack the academic qualifications to match into more lucrative specialties.

Political orientation was a strong predictor of medical student health policy attitudes. Self-described liberals were more than four times more likely to endorse a professional obligation to care for the under- and uninsured than their conservative counterparts. More strikingly, when compared to conservatives, liberals were more than six times more likely to favor limiting reimbursements to expand care to the underserved. Both of these findings demonstrate underlying values that predict political orientation, such as a commitment to equity and social welfare among liberals and a commitment to free market ideals among conservatives.

One important mechanism which may shape medical student attitudes is student debt, which continues to grow [18, 27]. In 2008, twenty-three percent of graduating allopathic medical students reported debt in excess of $200,000 which later increased to forty-six percent in 2014 [16, 17].

The reader should be aware of several limitations when interpreting our findings. This survey study invited all US accredited osteopathic and allopathic institutions and there was substantial variation in the level of response from different institutions and from different regions. This variation is also reflected in the overall national response rate of just over 2%. This could result in potential selection bias and limit the representativeness of our findings. However, all US medical schools were invited to participate. Furthermore, ours is the only study of which we are aware that has invited all accredited US medical schools in order to survey the health policy attitudes of medical students attending both US accredited allopathic and osteopathic medical institutions. Given the unique scope of this pilot study, it’s 2,355 perspectives provide insight into a more comprehensive evaluation of the US physician supply. As a result of survey self-administration, there may be possible selection bias. Furthermore, medical students with more extreme attitude positions may have been more likely to participate. To help address these limitations, we weighted our sample by class year and race to improve the representativeness of the findings. Additionally specialty preference may not accurately predict specialty choice. To limit this influence we provided the selection “undecided”, “primary care”, “surgery” and collapsed potential procedural and non-procedural subspecialty selections into “other”. Our findings compare favorably to extant literature about the policy attitudes of American medical students, showing medical students to generally support health reform. However, our findings are more detailed, focusing on the mediators of policy attitudes among medical students akin to studies of the physician literature. Our study assessed anticipated graduating debt burden in medical students prior to their graduation, rather than actual debt after graduation. As a result, the outcome of anticipated debt may not accurately approximate real debt burdens at the time of graduation. Nonetheless it is notable that our findings are consistent with current trends in medical student debt [17].

## Conclusions

With respect to future research, our findings raise several questions. First, it is unclear how attitudes may change throughout the training life course. In that respect, longitudinal studies are needed to track changes in provider attitudes through training-beginning in medical school, progressing through residency training, and extending into early practice. Second, our study was conducted during a unique time in the history of health policy in the United States. Future research is needed to understand the influence of ongoing changes in the health policy context on medical student attitudes into the future.

With respect to health policy, medical students and physicians both perceive an obligation to care for the under- and uninsured. However, modest differences in agreement between physicians and medical students indicate that medical students may be more likely to object to reimbursement cuts and the use of cost effectiveness data when compared to physicians in the literature. Those with high debt burdens were more likely to object to reimbursement cuts or the use of cost effectiveness data in decision-making, while those planning to pursue a career in primary care were particularly more likely to support these measures. As both of these are plausibly modifiable, policies that promote primary care and decrease educational debt may encourage bending of the healthcare cost curve [16].

## Supporting Information

S1 File Excluded Respondents. (XLSX)

S2 File Survey Instrument. (PDF)

S3 File Analysis of Political Orientation Similarity. (XLSX)

S4 File Dataset. (ZIP)
