# Supplementary material for: The Health Policy Attitudes of American Medical Students: A Pilot Survey
Source: PLoS One. 2015 Oct 16;10(10):e0140656. doi: 10.1371/journal.pone.0140656 (PMC4608797; doi:10.1371/journal.pone.0140656)
Supplement: S2 File — (PDF) [file pone.0140656.s002.pdf]

STONY BROOK
STATE UNIVERSITY OF NEW YORK
COMMITTEES ON RESEARCH INVOLVING HUMAN SUBJECTS
Established 1971

Exit this survey

*** What is your age in years?**

Less than 21 years of age

Between 21 and 24 years of age

Between 25 and 29 years of age

Between 30 and 34 years of age

35 years of age or greater

*** What is your gender?**

Male

Female

*** What is your self described race or ethnic group? Please check all that apply.**

White | Asian (also includes South Asian) | Black | American Indian or Alaska Native | Hispanic/Latino | Other

*** What level of medical school are you currently in?**

1st year medical student

2nd year medical student

3rd year medical student

4th year medical student

I never was nor am I currently a medical student

I graduated from medical school

Other

Other (please specify)

*** In which aspect of medicine are you most considering a career?**

Primary Care

Surgery

Procedural Specialty

Nonprocedural specialty

Nonclinical specialty

Other

Undecided

*** Were you or a loved one significantly affected by the recent economic downturn?**

Yes

No

*** What is your anticipated educational debt upon graduation?**

No educational debt

Less than $50,000

$50,000 - $100,000

$100,000 - $150,000

$150,000 - $200,000

$200,000 - $250,000

$250,000 - $300,000

Greater than $300,000

*** Which category of medical school do you currently attend?**

Public

Private

*** Please select the U.S. region in which your medical school resides.**

South

Midwest

Northeast

West

Outside the 50 states of the U.S.A.

*** Do you intend to practice medicine in the USA?**

Yes

No

Undecided

*** Which form of employment are you most considering as a future physician?**

Self Employment / Private Practice

Hospital Employment

Other

Undecided

*** What is your political self-characterization?**

Moderate

Liberal

Conservative

Other

*** Addressing societal health policy issues, as important as that may be, falls outside the scope of my future professional obligations as a physician.**

Strongly Disagree

Moderately Disagree

Moderately Agree

Strongly Agree

✱ Every physician is professionally obligated to care for the uninsured and the underinsured.

Strongly Disagree

Moderately Disagree

Moderately Agree

Strongly Agree

✱ I would favor limiting reimbursement for expensive drugs and procedures if that would help expand access to basic health care for those currently lacking such care.

Strongly Disagree

Moderately Disagree

Moderately Agree

Strongly Agree

✱ I would favor limiting reimbursement and the ability to repay student loans if that would help expand access to basic health care for those currently lacking such care.

Strongly Disagree

Moderately Disagree

Moderately Agree

Strongly Agree

✱ Indicate the degree to which you object (if at all), for moral reasons to the following practice- Using cost-effectiveness data to determine which treatments will be offered to patients.

No Moral Objection

Moderate Moral Objection

Strong Moral Objection

✱ Of the following entities, which do you feel is the most responsible for advocating for your professional interests?

Professional Organizations (Ex: American Medical Association, American Association of Medical Colleges)

Student organizations (Ex: American Medical Student Association, AMA Medical Student Section)

Medical Schools

Students

Myself

Optional - Please write in and or select a total of 5 issues related to US health policy you feel are most important.

Overuse of tests and procedures that are unwarranted and or have limited evidence of effectiveness

Culture of luxuries before necessities

Emergency room misuse

Limited prescription of generic drugs

Increasing scope of practice of mid-level health care providers (NP, PA, midwife etc.)

Increasing consumer/patient expectations

For-profit insurance / managed health care

Medical student indebtedness / The rising cost of medical school tuition

Limited use of preventative medicine / Limited investment in public health

Increasing number of uninsured and underinsured

Increase in the number of medical students without comparable increase in residency slots

Doctor shortage

Limited medical malpractice reform

Limited Medicaid and Medicare reimbursement

Increasing bureaucracy / Increasing administrative costs

Limited patient health education / health literacy

Media mischaracterization

Mandatory acceptance of Medicare/Medicaid as a possible term of licensure

Healthcare disparities

Other (When writing please separate issues with "/". You may write up to 250 characters.)

Optional - Please enter the valid MEDICAL SCHOOL email address at which you wish to receive your $100 gift certificate should you be a winner. Again, this is optional and is strictly to provide a means of incentive disbursement. Your response to this question will be destroyed at the completion of the study and

will remain confidential until that point. You will not be contacted by an investigator unless you are a $100 visa gift certificate winner.

Optional - please feel free to submit comments (up to 1000 characters in length). Thank you for taking part in this survey.

Prev Done

Powered by **SurveyMonkey**
Check out our sample surveys and create your own now!
